# TG‐51: Experience from 150 institutions, common errors, and helpful hints

**DOI:** 10.1120/jacmp.v4i2.2524

**Published:** 2003-03-01

**Authors:** R. C. Tailor, W. F. Hanson, G. S. Ibbott

**Affiliations:** ^1^ Department of Radiation Physics University of Texas M.D. Anderson Cancer Center 1515 Holcombe Blvd., Box 547 Houston Texas 77030

**Keywords:** TG‐51, calibration protocol, dosimetry, plane‐parallel chamber

## Abstract

The Radiological Physics Center (RPC) is a resource to the medical physics community for assistance regarding dosimetry procedures. Since the publication of the AAPM TG‐51 calibration protocol, the RPC has responded to numerous phone calls raising questions and describing areas in the protocol where physicists have had problems. At the beginning of the year 2000, the RPC requested that institutions participating in national clinical trials provide the change in measured beam output resulting from the conversion from the TG‐21 protocol to TG‐51. So far, the RPC has received the requested data from ~ 150 of the ~ 1300 institutions in the RPC program. The RPC also undertook a comparison of TG‐21 and TG‐51 and determined the expected change in beam calibration for ion chambers in common use, and for the range of photon and electron beam energies used clinically. Analysis of these data revealed two significant outcomes: (i) a large number (~ 1/2) of the reported calibration changes for photon and electron beams were outside the RPC's expected values, and (ii) the discrepancies in the reported versus the expected dose changes were as large as 8%. Numerous factors were determined to have contributed to these deviations. The most significant factors involved the use of plane‐parallel chambers, the mixing of phantom materials and chambers between the two protocols, and the inconsistent use of depth‐dose factors for transfer of dose from the measurement depth to the depth of dose maximum. In response to these observations, the RPC has identified a number of circumstances in which physicists might have difficulty with the protocol, including concerns related to electron calibration at low energies $\left(R_{50}<2\;\text{cm}\right)$, and the use of a cylindrical chamber at 6 MeV electrons. In addition, helpful quantitative hints are presented, including the effect of the prescribed lead filter for photon energy measurements, the impact of shifting the chamber depth for photon depth‐dose measurements, and the impact of updated stopping‐power data used in TG‐51versus that used in TG‐21, particularly for electron calibrations.

PACS number(s): 87.53.–j, 87.66.–a

## INTRODUCTION

In 1999, the AAPM published a calibration protocol based on an absorbed‐dose‐to‐water calibration standard.^1^ This calibration protocol, known as TG‐51, contains a number of procedures and concepts that distinguish it from the older TG‐21 protocol.^2^ For example, TG‐51 introduces parameters such as $k_{Q}$ and $k_{\text{ecal}}$, the phantom material is limited to liquid water, the reference depth for electron calibration is not $d_{\max}$, and photon‐beam energy is specified in terms of photon‐only percent depth dose, which requires measurements with a lead filter placed in high energy beams.

The Radiological Physics Center (RPC) is charged by the NCI with monitoring the physics dosimetry and quality assurance activities at megavoltage therapy facilities that participate in NCI funded cooperative clinical trials. The RPC currently monitors approximately 1300 such facilities. As part of its responsibility, the RPC conducts audits of dose calibration of treatment units in use at these facilities. The RPC is therefore interested in the experiences of institutions as they implement the TG‐51 protocol, and interested in assisting them when problems or questions arise.

The RPC was involved in beta testing the TG‐51 protocol,^3^ and implemented the protocol on January 1, 2000. The RPC also mailed a set of forms to the participating institutions. These forms outlined a set of procedures to document the magnitude of the change in the dose to the patient as a result of converting from the previous TG‐21 protocol to the TG‐51 protocol, and to report that change to the RPC. The institutions were asked to indicate the date they expected to convert to the TG‐51 protocol, and were advised to contact the RPC before implementing the change if the change was ⩾2.5%. To date, the RPC has received the requested data from about 150 institutions reporting measurements for more than 1200 photon and electron beams. These data have been analyzed and institutions contacted when unexplained discrepancies were found. Members of the RPC also presented numerous lectures and workshops on the TG‐51 protocol and its implementation. The RPC therefore received feedback from many sources on the implementation of the new protocol.

In this paper, we intend to summarize what we have learned that can help other institutions converting to the TG‐51 protocol. Many of the issues are interrelated; however, we have tried to separate and categorize them. We begin by presenting results of the data from the 150 institutions, subsequently summarizing possible sources of the discrepancies between what the RPC expected and what the institutions report. Many of these discrepancies exceed 2%. We then discuss issues specific to TG‐51 pertaining to: (i) the use of plane‐parallel chambers, (ii) both electrons and photons, (iii) photons only, and (iv) electrons only. Some issues are merely mentioned, some are discussed in more detail, and others are supported with data.

## METHODS

Our knowledge of problems, confusions, and complications introduced by the TG‐51 protocol is based on our own research and on feedback from many sources. The RPC has presented posters and papers on several aspects of TG‐51^3^ ^,^ ^4^ and discussed a wide spectrum of questions from the medical physics community. The data provided by institutions on the RPC forms were reviewed and the participating physicists were contacted whenever the RPC questioned the data. We also learned from our own false starts and confusions trying to implement TG‐51 in the RPC mailed‐TLD and on‐site dosimetry review programs.

Tailor and Hanson^4^ computed the expected ratio of the doses determined according to TG‐51 and TG‐21. These calculations were performed to simulate measurements with a variety of ionization chambers, and most photon and electron beams in common clinical use. These ratios range from 1.000 in an 18 MV photon beam up to 1.025 in a 20 MeV electron beam. These ratios are referred to in this paper as the “RPC‐expected ratios.” The forms provide both the magnitude and direction of the change in dose delivery to patients due to implementing TG‐51. The ratio of doses measured by the institution for the two protocols (the TG‐51/TG‐21 ratio) was compared to the RPC‐expected ratio. If the institution's ratio differed from the RPC‐expected ratio by more than 1%, an independent calculation by the RPC was triggered. The Tailor and Hanson paper^4^ eliminates many of the sources of discrepancy discussed in this paper, including the influence of the ADCL calibrating the institution's instrument. Therefore, this 1% criterion was intended to detect as many discrepancies as possible. If the RPC's independent determination of dose by both TG‐51 and TG‐21 did not clarify the discrepancy, the participating physicist was contacted for discussion and clarification. Through this review, the RPC was able to uncover and understand various sources of error, inconsistency, and uncertainty in beam output calculations.

## RESULTS AND DISCUSSION

### A. Data from institution questionnaires

**Figure 1:**
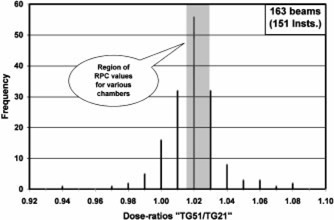
Ratio of dose calculated by TG‐51 vs the dose calculated by TG‐21 for 163 beams from 151 institutions for electrons with nominal energies of 18 MeV or more. The hatched area represents the ratios expected by the RPC for these energies for most chambers in clinical use.

**Figure 2:**
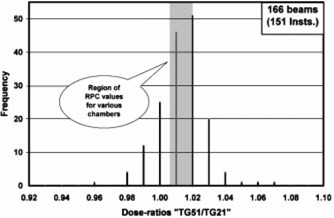
Ratio of dose calculated by TG‐51 vs the dose calculated by TG‐21 for 166 beams from 151 institutions for electrons with nominal energies of 6 MeV or less. The hatched area represents the ratios expected by the RPC for these energies for most chambers in clinical use.

**Figure 3:**
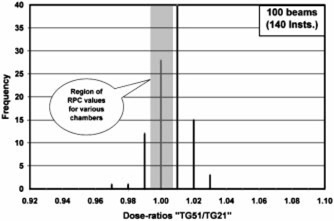
Ratio of dose calculated by TG‐51 vs the dose calculated by TG‐21 for 100 beams from 151 institutions for photons with nominal energies of 18 MV or more. The hatched area represents the ratios expected by the RPC for these energies for most chambers in clinical use.

**Figure 4:**
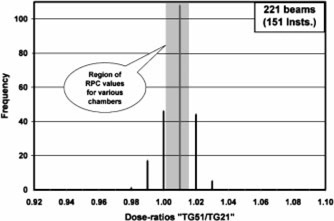
Ratio of dose calculated by TG‐51 vs the dose calculated by TG‐21 for 221 beams from 151 institutions for photons with nominal energy of 6 MV. The hatched area represents the ratios expected by the RPC for these energies for most chambers in clinical use.

The TG‐51/TG‐21 dose ratios for 1230 electron and photon beams received from the first 151 institutions have been compared against the RPC‐expected values generated in the earlier paper.^4^ The results are presented in Figs. 1–4 as frequency histograms of the TG‐51/TG‐21 dose ratios. Only low and high‐energy beams for both photons and electrons are presented to provide an overview of the spectrum of energy and modality. Figures 1 and 2 correspond to high (18–22 MeV) and low (5–6 MeV) energy electron beams, respectively. Similarly, Figs. 3 and 4 correspond to high (18–24 MV) and low (6 MV) energy photon beams, respectively. The shaded region in each figure indicates the range of RPC‐expected values for a wide selection of cylindrical and plane‐parallel ion chambers in common use. The average of the institution data and the RPC‐expected values match very well for all beams except for 18 MV x rays, which show a 1% difference. The origin of this difference is unclear. The RPC range has a spread of no more than ±0.7% for any beam energy, while the range of the institutions’ data is very wide (e.g., from

0.93−1.06 for 18–22 MeV electrons). A large number (~1/2) of the reported ratios differed from the RPC‐expected values by more than 1%. Moreover, there are a reasonable number of electron beams showing discrepancies of 3% or more, including a few serious outliers. It should be noted that the institution data even inside the shaded area may be found to disagree with the RPC by >1% once the specific chamber is identified.

One of the RPC's roles is to help institutions by finding the origin of the disagreement, and to help the institution to resolve the difference. To assist in this endeavor, the RPC requested and reviewed TG‐51 and TG‐21 output calculations from the institutions concerned. The review process helped to identify the sources of discrepancy, and resolution was often achieved through communication with the institutions. A number of sources of discrepancies were discovered. They consist of inconsistencies, uncertainties, confusions, and errors, which we have listed below as a guide to others trying to implement TG‐51. We have made no attempt to quantify the relative frequency of the various issues, but all have been experienced a number of times.

### B. Sources of discrepancies on the forms

1. Measurement at different depths for the two protocols: Photon or electron output calibration using TG‐21 and TG‐51 requires ionization measurements at different depths. This results in two sources of uncertainty. The measurement uncertainty enters twice, and any inconsistency in depth‐dose data also contributes to the discrepancy.
2. Use of different $P_{\text{ion}}$ and $P_{\text{pol}}$ values for the two protocols: These values should be the same for both TG‐21 and TG‐51, except possibly for small changes due to measurement at different depths.
3. Setup uncertainties and machine drift: To best determine the actual change in dosimetry between the two protocols, both sets of measurements should be made close in time and with an undisturbed setup.
4. Use of different phantom materials for the two protocols: TG‐51 requires the phantom material to be liquid water, but many people had used plastic (including various “water equivalent” plastics) for TG‐21 measurements. The use of plastic phantoms has been shown to yield dose determinations different from liquid water.^5^
5. Use of different ion chambers for the two protocols: Any difference in the NIST‐traceable calibration would be reflected in the measurements. Also, model‐to‐model differences in ion chambers may not be ideally accounted for by both protocols.
6. Use of plane‐parallel chambers: Measurements^6^ have shown that the selection of calibration techniques can introduce uncertainties in excess of 2% (several of these issues are discussed below).
7. The use of different depth dose data for the two protocols: A depth dose correction is required to transfer dose from the reference depth to $d_{\max}$. For photons it is the question of whether or not the $0.6r_{\text{cav}}$ shift is incorporated. For electrons it is the question of whether the electron stopping‐power data used are from TG‐51 (Burns *et al.* ^7^) or TG‐21 tables (this is discussed later).
8. There is confusing labeling of several chambers in the $k_{R50}^{\prime }$ figures: (i) PR‐06C/G chambers are identified with two groups of chambers in Fig. 5. (ii) Chambers NE2581 and N30004 are identified with one group of chambers in Fig. 5 and a different group in Fig. 7. Review with Rogers,^8^ the author of the original data used to generate these figures, indicates: (i) in Fig. 5, the correct grouping of PR06C/G chambers is with the NE2571, N23331, and NE2581 chambers. (ii) Chamber identification in Figs. 5 and 7 is based on the best grouping appropriate for that figure without regard to the other figure, so crossover of chambers is correct.
9. Errors in reading tables and figures, and in selection of protocol factors.

### C. Issues relating to plane‐parallel chambers

Tailor and Hanson^4^ discussed at length the problems arising from the choice of calibration technique for plane‐parallel chambers. Physicists must compare with a cylindrical chamber in a high‐energy electron beam or apply an ADCL calibration in conjunction with either $k_{\text{ecal}}$ from TG‐51^1^ or $N_{\text{gas}}/N_{X}$ from TG‐39^9^ or the literature.^10^ There are two separate issues: (i) Using an ADCL calibration with $k_{\text{ecal}}$ values from the TG‐51 protocol can result, for some plane‐parallel chambers, in a dose error of up to 2%. (ii) Mixing the two chamber‐calibration techniques can introduce differences in the dose ratio, TG‐51/TG‐21, of up to 4% for these chambers.

We emphasize the recommendation from TG‐51 that the product $\left(N_{D,w}^{60_{\text{Co}}}\cdot k_{\text{ecal}}\right)$ be carefully determined by comparison with an ADCL‐calibrated cylindrical chamber in a high‐energy electron beam. We also recommend that an ADCL calibration be obtained for the plane‐parallel chamber and $\left(N_{D,w}^{60_{\text{Co}}}\cdot k_{\text{ecal}}\right)$ be determined using values of $k_{\text{ecal}}$ from TG‐51. If the difference in the products determined by the two methods is 1% or less, either value of $\left(N_{D,w}^{60_{\text{Co}}}\cdot k_{\text{ecal}}\right)$ be determined using values of $k_{\text{ecal}}$ may be used. However, if the difference exceeds 1%, the cross‐calibration method should be used to maintain consistency with photon beam calibrations.

### D. Issues pertaining to both electrons and photons

1. Influence of ADCL: Due to uncertainties in the standards for $N_{D,w}^{60_{\text{Co}}}$ be determined using values of $k_{\text{ecal}}$, and $N_{X}$, and in the distribution of these factors to the users, the values of $N_{D,w}^{60_{\text{Co}}}$ and $N_{X}$, or the ratio of the two, vary from one ADCL to another, introducing a discrepancy (⩽ 1%) with the RPC‐expected TG‐51/TG‐21 dose ratios. Tailor and Hanson^4^ discuss this discrepancy and present TG‐51/TG‐21 dose ratios with the influence of calibration factor removed.
2. Where to place the axis of a cylindrical chamber: It is important to note that the TG‐51 protocol is consistent for both modalities.
  - Depth dose: Whenever depth dose (clinical depth dose or beam quality specification) is measured, the shift to the effective point of measurement is used.
  - Beam output calibration: When measuring $M_{\text{raw}}$ for calibration, the center (axis) of a cylindrical chamber is placed at the reference depth ($d_{\text{ref}}$ for electrons and 10.0 cm for photons). There is no physical shift used; the concept of an effective point of measurement is accounted for in the calculation ($P_{\text{gr}}$ is explicitly included for electrons and $P_{\text{repl}}$ is implicitly included in $k_{Q}$ for photons). For a plane‐parallel chamber, the measurement point is the inner surface of the front window.
3. $P_{\text{pol}}:$ Be sure to include the sign of the reading in the equation for $P_{\text{pol}}$ (equation 9 of the protocol). Since the polarity effect is small at calibration depths, $P_{\text{pol}}$ is close to unity. A very small value of $P_{\text{pol}}\;(<10^{-2})$ indicates a calculation problem with signs.
4. $P_{\text{ion}}:$
  - $P_{\text{ion}}$ depends on the dose per pulse. Therefore, it is a function of depth so it should be measured at the relevant depth. In addition, there are several linacs with high dose rate electron capabilities (e.g., the Varian Clinac CD and EX and the Siemens ME). The $P_{\text{ion}}$ correction for electrons on these units is significantly larger than on other linacs.
  - On some linacs, the dose per monitor unit varies with the dose rate set on the console.
  - When measuring $P_{\text{ion}}$ with the half voltage technique for pulsed beams, if the ratio of $M^{H}/M^{L}$ (equation 12) is less than 1.02 $P_{\text{ion}}=M^{H}/M^{L}$ to within 0.1%. This makes a convenient redundant check of $P_{\text{ion}}$.
  - There is a typographical error on worksheet A, item 8 (page 1862) for the Co‐60 $P_{\text{ion}}$ formula. Readers should use equation 11 on page 1854 of the protocol.
5. $P_{\text{ion}}$ and $P_{\text{pol}}$:
  - $P_{\text{ion}}$ and $P_{\text{pol}}$ are specific to a combination of ion‐chamber, linac, beam modality, and beam energy. As long as the combination stays the same, $P_{\text{ion}}$ and $P_{\text{pol}}$, in principle, should remain the same.
  - To measure $P_{\text{ion}}$ and $P_{\text{pol}}$ well is both nontrivial and time consuming. To obtain a meaningful value, readings should be repeated at each polarity and/or potential until reproducible nontrending charge measurements are obtained. It is also a good idea to return to the original polarity/potential to assure that these values reproduce.
  - Therefore, it is more important to measure $P_{\text{ion}}$ and $P_{\text{pol}}$ carefully than to measure them frequently.
6. Several currently available chamber models are not explicitly included in TG‐51. Section XI of the protocol describes a procedure for identifying a listed chamber whose values of $k_{Q},k_{\text{ecal}}$, and $k_{R50}$ are expected to be similar. The materials of the thimble and collector are most important, while the exact dimensions are less critical. As an example, the published parameters for the PTW N30001 (N23333) can also be used for the PTW N30006 chamber.

### E. Issues pertinent to photon beams

1. Beam Quality:
  - a. The beam‐quality specifier, ${\%\text{dd}(10)}_{x}$, being a depth dose parameter, requires a shift to the effective point of measurement. The center of the chamber is placed at $10.0+0.6r_{\text{cav}}$.
  - ${\%\text{dd}(10)}_{x}$ must be measured at 100 cm SSD regardless of the nominal SSD of the therapy unit.
  - ${\%\text{dd}(10)}_{x}$ represents the depth dose at 10 cm depth without the influence of electron contamination from the collimator. For photon energies of 10 MV or more, electron contamination at $d_{\max}$ is significant and must be corrected for. TG‐51 chose to replace the unknown contamination with a known contamination from 1 mm of Pb, for which corrections have been determined by Monte Carlo calculations. Therefore a sheet of lead, thickness 1 mm±0.2 mm, is required when determining the ${\%\text{dd}(10)}_{x}$ of photon energies of 10 MV or greater. The lead must be the last solid material between the target and the water surface, and must be removed for calibration and all other measurements.
  - The protocol includes an “interim alternative” (equation 15) to determine ${\%\text{dd}(10)}_{x}$ without the use of lead. The RPC measured both ${\%\text{dd}(10)}_{\text{Pb}}$ and ${\%\text{dd}(10)}_{o}$ (with and without the Pb filter) on all machines reviewed. From these data the error in $k_{Q}$, and hence in output, resulting from the use of the “interim alternative” (no Pb) has been determined. Typical data are presented in Table I.
  - Equations (13) or (14) of the protocol are used to convert ${\%\text{dd}(10)}_{\text{Pb}}$ into ${\%\text{dd}(10)}_{x}$. On several occasions known to us, physicists have used fractional depth dose, ${\text{fdd}(10)}_{\text{Pb}}$, rather than percent depth dose ${\%\text{dd}(10)}_{\text{Pb}}$ in the additive terms of these equations. The derived ${\%\text{dd}(10)}_{x}$ should be no more than 2.5% higher than the measured ${\%\text{dd}(10)}_{\text{Pb}}$. The erroneous use of ${\text{fdd}(10)}_{\text{Pb}}$ results in differences near 20%.
Clinical depth‐dose data:
  - TG‐51 recommends the use of a shift to the effective point of measurement in the determination of depth dose data. TG‐21 did not use a shift; however, it did call for a $P_{\text{repl}}$ value of unity at $d_{\max}$ and a nonunity $P_{\text{repl}}$ value at other depths. For a Farmer‐like chamber, $P_{\text{repl}}$ varies from 0.991 at ${}^{60}\text{Co}$ to 0.994 at high energies. The result is that TG‐51 shifted depth dose is essentially equal (⩽0.2%) to TG‐21 unshifted depth‐ionization data corrected for $P_{\text{repl}}$ at depths other than $d_{\max}$.
  - The gradient of photon depth dose data is nearly constant past $d_{\max}$. Therefore, neither the use of a depth shift, nor application of $P_{\text{repl}}$, will significantly affect the gradient. Most tumors treated with photons are located significantly deeper than $d_{\max}$. If depth‐dose data are normalized at a clinically relevant depth, the use of a depth shift or application of $P_{\text{repl}}$ will result in differences only near $d_{\max}$. Reference calibration at 10 cm depth provides the needed normalization. This implies that tumor doses are virtually independent of the various methods of depth dose measurement.
  - Therefore, if one is willing to accept additional small uncertainties near $d_{\max}\;(<1\%)$, it may not be necessary to recommission photon beams to incorporate the shift recommended in TG‐51.
  - Absorbed dose determined at the reference depth for calibration (10 cm) must be converted to dose at the reference depth for the treatment‐planning system (TPS) (frequently $d_{\max}$. As recommended by TG‐51, in order for the dose at tumor depth to be consistent, the clinical depth dose data at 10.0 cm (the same as used in the TPS) must be used for this conversion. Do not use ${\%\text{dd}(10)}_{x}$ or the clinical depth‐dose data stated at 10.2 cm.
Protective cap:
  - TG‐51 recommends an acrylic (PMMA) cap of wall thickness < 1 mm, and wall‐to‐cap air gap ⩽0.2 mm for nonwaterproof chambers. Questions have been raised about the effect of this cap. Hanson and Tinoco^11^ report that 1 mm of acrylic should introduce no more than 0.2% uncertainty.
  - The use of latex condoms is also discussed in TG‐51, with the caution to remove any excess talcum powder to avoid problems with the chamber behavior.

**Table I:** Error in $k_{Q}$ if lead sheet is not used.

	Nominal MV	${\%\text{dd}(10)}_{\text{Pb}}@30$	$\%\text{dd}(10){|}_{0}^{\text{Pb}@30}$	${\%\text{dd}(10)}_{x}{|}_{0}^{\text{Pb}@30}$	$k_{Q}{|}_{0}^{\text{Pb}}$
Varian machines	23	80.0	1.010	1.018	1.002
	18	79.7	1.008	1.015	1.002
	10	73.4	1.004	1.009	1.001

### F. Issues pertinent to electron beams

1. Calibration:
  - Unlike depth‐dose data, there is no physical shift to the effective point of measurement for calibration measurements. The axis of a cylindrical chamber or the inner surface of the entrance window of a plane‐parallel chamber is set at $d_{\text{ref}}$.
2. Electron depth‐dose data:
  - When measuring electron %dd with a cylindrical chamber, a shift of $+0.5r_{\text{cav}}$ is used at all depths. This shift was also prescribed by the AAPM TG‐25 report.^12^ This shift does not apply to plane‐parallel chambers.
  - To convert $D_{\text{ref}}$ to $d_{\max}$, the dose rate at $d_{\text{ref}}$ must be divided by the fdd at $d_{\text{ref}}$. As with photons, it is important that the clinical depth‐dose data be used.
  - TG‐51 recommends incorporating updated stopping power ratios from Burns *et al.* ^7^ This impacts on output at $d_{\max}$ because it changes the depth‐dose data. Physicists continue to ask us what the impact would be if they did not convert their clinical depth dose data to incorporate the Burns stopping power data. Table II shows that the impact on dose at $d_{\max}$ (expressed as fdd at $d_{\text{ref}}$ is less than 0.5% over the range of clinical electron beams. Therefore, recommissioning of clinical data to incorporate the updated stopping power data may not be necessary.
3. Several issues regarding the determination of $P_{\text{gr}}$ should be considered:
  - The value of $P_{\text{gr}}$ can be >1, because at low beam energies (⩽6 MeV), $d_{\text{ref}}$ can be in the build‐up region.
  - Since $P_{\text{gr}}$ is a direct multiplier of $M_{\text{raw}}$, measurements at $\left(d_{\text{ref}}+0.5\;r_{\text{cav}}\right)$ should have the same desired level of precision as $M_{\text{raw}}$.
4. As discussed above under general issues, the dose per pulse for the Varian CD and EX models is twice that of the C models, leading to higher $P_{\text{ion}}$ values. As an example, $P_{\text{ion}}$ for an NEL2571 ion chamber is typically 1.016 for electron beams from CD or EX models versus 1.008 for electrons from C model linacs.
5. Use of cylindrical chambers: The RPC is frequently asked if a plane‐parallel chamber is mandatory for ⩽ 6 MeV electron calibration, and what error results if a cylindrical chamber is used. At the present time, TG‐51 requires the use of a plane‐parallel chamber for low energy electron calibration (⩽6 MeV). However, the RPC has encouraged Task Group 51 to consider extending the lower limit for cylindrical chambers to $R_{50}$ near or below 2.0 cm.
6. $k_{R50}^{\prime }$ values for low energy electrons: TG‐51 provides values of $k_{R50}^{\prime }$ for electron beams with $R_{50⩾}2.0\;\text{cm}$. There are a number of clinical electron beams in use with $R_{50}<2.0\;\text{cm}$. Tailor and Hanson have shown that equations 19 and 20 of TG‐51 (for Farmer‐type and well guarded plane‐parallel chambers, respectively) can be extrapolated to $R_{50}=1.0\;\text{cm}$, to obtain values of $k_{R50}^{\prime }$ without the introduction of significant error^4^
7. Clinical depth‐dose data: The RPC is aware that some institutions use percent ionization as their clinical depth‐dose data, rather than correcting ionization to dose using TG‐25.^12^ At high energies, this can introduce errors of 2–3 % in the depth‐dose value at $d_{\text{ref}}$, which will be reflected as a discrepancy with the RPC‐expected TG‐51/TG‐21 dose ratios.

**Table II:** Impact of electron stopping powers (TG‐51 vs TG‐21) on TG‐51 output calibration at $d_{\max}$ due to differences in depth‐dose factor at $d_{\text{ref}}$. Data are provided for typical beam characteristics.

Nominal energy (MeV)	Beam characteristics		Effective depth (cm)	${\bar{L/}p)}_{\text{air}}^{\text{water}}$		Stopping powers $d_{\text{ref}}$ vs $d_{\max}$ ^b^		Ratio of fdd at $d_{\text{ref}}$ TG‐51^a^ vs TG‐21	
	$E_{0}$ (MeV)	$R_{50}$ (cm)		TG51^a^	TG21	TG51^a^	TG21		
5	4.59	1.96	$d_{\max}$	$1.0_{5}$	1.078	1.088	‐	‐	‐
			$d_{\text{ref}}$	$1.1_{4}$	1.082	1.090	1.004	1.002	1.002
12	10.91	4.76	$d_{\max}$	$2.8_{0}$	1.047	1.051	‐	‐	‐
			$d_{\text{ref}}$	$2.7_{5}$	1.046	1.050	0.999	0.999	1.000
20	19.15	8.40	$d_{\max}$	$2.0_{0}$	0.980	0.978	‐	‐	‐
			$d_{\text{ref}}$	$4.9_{5}$	1.019	1.021	1.039	1.044	0.996

a Data from Burns *et al.* ^7^

b ${\bar{L/}p)}_{\text{air}}^{\text{water}}$ at $d_{\text{ref}}÷{\bar{L/}p)}_{\text{air}}^{\text{water}}$ at $d_{\max}$.

## CONCLUSIONS

The ratios of absorbed dose as determined by the TG‐51 and TG‐21 protocols have been calculated for a variety of cylindrical and plane‐parallel chambers over a range of photon and electron energies.^4^ This analysis is also available on the RPC web‐site (http://rpc.mdanderson.org). Readers are encouraged to compare their measured TG‐51/TG‐21 dose ratios with these data. In this paper we have identified a number of issues that could result in discrepancies between the readers and the RPC‐expected dose ratios. Some of them are minor differences due only to uncertainties in dosimetry, and therefore irresolvable; however, some are due to errors or confusions, which can be corrected. This paper discusses primarily those discrepancies introduced by improper implementation of TG‐51, but does not discuss, in any detail, uncertainties and errors introduced by improper implementation of TG‐21. If the issues discussed in this paper have been considered, and the measured TG‐51/TG‐21 dose ratios are still significantly different than the RPC‐expected ratios, the reader should contact the RPC before implementing TG‐51. The RPC physicist will review calculations of both TG‐51 and TG‐21 to help identify the origin of the discrepancy, its impact on patient dosimetry, and possible resolutions.

## ACKNOWLEDGMENTS

The RPC wishes to thank the many viewers of the posters and attendees at lectures and workshops who provided much constructive feedback. The authors wish to acknowledge the other physicists in the RPC who were involved in the workshops, in the review of institution's calculations, and in phone discussions with physicists. We also wish to recognize Cindy Davis, a dosimetrist at the RPC, for compilation of the institution data. This work was supported by PHS Grant No. CA10953 awarded the NCI, DHSS.
